# Toward reproducible pig gut microbiome profiling through standardized methodologies

**DOI:** 10.1093/ismeco/ycag097

**Published:** 2026-04-11

**Authors:** Timur Yergaliyev, Samuel O Enokela, Gabi Eberhardt, Krzysztof Flisikowski, Stéphanie C Hornburg, Henry Reyer, Jens Tetens, Klaus Wimmers, Jürgen Zentek, Amélia Camarinha-Silva

**Affiliations:** Hohenheim Center for Livestock Microbiome Research (HoLMiR), University of Hohenheim, 70599 Stuttgart, Germany; Institute of Animal Science, University of Hohenheim, 70599 Stuttgart, Germany; Hohenheim Center for Livestock Microbiome Research (HoLMiR), University of Hohenheim, 70599 Stuttgart, Germany; Institute of Animal Science, University of Hohenheim, 70599 Stuttgart, Germany; Hohenheim Center for Livestock Microbiome Research (HoLMiR), University of Hohenheim, 70599 Stuttgart, Germany; Institute of Animal Science, University of Hohenheim, 70599 Stuttgart, Germany; Infection Pathogenesis, Technical University of Munich, 85354 Freising, Germany; Institute for Animal Nutrition and Physiology, Christian-Albrechts University Kiel, 24118 Kiel, Germany; Physiological Genomics, Research Institute for Farm Animal Biology (FBN), 18196 Dummerstorf, Germany; Functional Breeding, Georg-August University, 37077 Göttingen, Germany; Physiological Genomics, Research Institute for Farm Animal Biology (FBN), 18196 Dummerstorf, Germany; Institute of Animal Nutrition, Freie Universität Berlin, 14195 Berlin, Germany; Hohenheim Center for Livestock Microbiome Research (HoLMiR), University of Hohenheim, 70599 Stuttgart, Germany; Institute of Animal Science, University of Hohenheim, 70599 Stuttgart, Germany

**Keywords:** microbiome, pigs, 16S rRNA gene amplicon sequencing, metagenome

## Abstract

Reproducible microbiome profiling is essential for linking microbial communities to host health, yet methodological variation continues to undermine reproducibility across studies. This problem is acute in pig microbiome research, where no standardized DNA extraction protocols exist despite the species’ importance in agriculture and biomedicine. Here, we benchmark how 12 widely used extraction kits influence microbiome outcomes in 16S rRNA gene amplicon sequencing and shotgun metagenomics of pig fecal samples. We demonstrate that extraction choice biases 16S rRNA gene datasets, affecting DNA yield, diversity, community composition, and spike-in recovery, whereas metagenomic taxonomy and functional profiles are comparatively robust. Kit-dependent recovery of Gram-positive versus Gram-negative taxa revealed systematic biases with direct consequences for biological interpretation. By integrating spike-in controls, taxonomic resolution, and metagenome-assembled genomes, we establish a framework for evaluating DNA extraction methods in animal microbiome research. Our findings demonstrate that 16S rRNA gene amplicon sequencing is more susceptible to extraction-driven artifacts than metagenomics, highlighting the need for standardized protocols to ensure reproducibility and comparability across pig microbiome studies. Moreover, while shotgun metagenomics was comparatively robust to DNA extraction choice, the number of assembled good-quality metagenome-assembled genomes recovered was strongly dependent on the extraction kit selection.

## Introduction

The gut microbiome plays a fundamental role in host physiology, health, and disease, and its study increasingly relies on high-throughput sequencing. However, methodological variation, particularly in DNA extraction, remains a major barrier to reproducibility across microbiome studies [1–3]. DNA extraction is a multi-step procedure, and each step can introduce variability depending on the protocol and reagents used [1].

Protocols that utilize mechanical lysis, such as bead-beating, recover Gram-positive bacterial taxa more efficiently, including *Bacillota* and *Actinomycetota* [4]. However, variations in bead sizes and materials further affect lysis efficiency [1]. Therefore, the bead-beating step is often the main factor affecting the recovered microbial community [5–7].

Although automated DNA extraction platforms provide higher throughput, they introduce additional variability and yield lower DNA concentrations and quality than manual methods. Despite being labor-intensive, manual methods can achieve superior results [8, 9].

Given that the major goals of gut metagenomic studies are to accurately characterize microbial composition, describe functional properties of identified taxa, and assess genetic heterogeneity [10], both metataxonomic and shotgun metagenomic (MG) approaches are employed. Targeted amplicon sequencing of the 16S rRNA gene hypervariable regions is a widely used metataxonomic strategy [11], even when sequencing depth is relatively low [12]. The sequencing output from this approach is grouped into clusters of similar sequences, such as Operational Taxonomic Units (OTUs), which underestimate diversity, dominance, and evenness metrics [13], or Amplicon Sequence Variants (ASVs) [10]. The choice of the 16S rRNA gene region and the primer combination used for amplification introduces systematic biases in taxonomic profiling [14–17].

In porcine studies, these hypervariable regions showed different performance across sample sets. The V4 region provides the most consistent characterization of bacterial and archaeal communities, while the V6–V8 region tends to overrepresent specific taxa. In swine fecal samples, the V4 region yields higher phylogenetic diversity compared to other regions. The V1–V3 region, however, reports a higher proportion of the phylum *Bacillota* and the genus *Blautia*, but underrepresents *Bifidobacterium* compared to the V3–V4 and V4 regions [18]. This discrepancy largely reflects primer bias, as the commonly used V1–V3 forward primer (27F 5′-AGAGTTTGATCCTGGCTCAG-3′) under-amplifies *Bifidobacterium* unless it is specifically modified [19, 20].

MG sequencing is more expensive but provides a quantitative overview of the entire genomic content, higher taxonomic resolution, and improved access to functional genes [21]. In porcine microbiome studies, MG sequencing identified more genera and microbial species than 16S rRNA gene amplicon sequencing [22]. Nevertheless, MG sequencing is limited by the substantial computational resources required [23] and by technical challenges such as chimeric contigs and misassemblies that can introduce biases [24]. Moreover, MG sequencing can be problematic in samples with high host or feed DNA content and a relatively low microbial DNA fraction [25].

In pigs, an animal model of major agricultural and biomedical relevance, DNA extraction protocols remain unstandardized, limiting reproducibility and cross-study comparability. To advance reproducible methodologies, this study investigates the impact of various DNA extraction kits on the bacterial community recovered from porcine fecal samples. We further examine how these effects vary across commonly targeted 16S rRNA gene regions and compare them with profiles obtained from MG sequencing of the same samples.

## Materials and methods

### Experimental animals and sample collection

**Figure 1:**
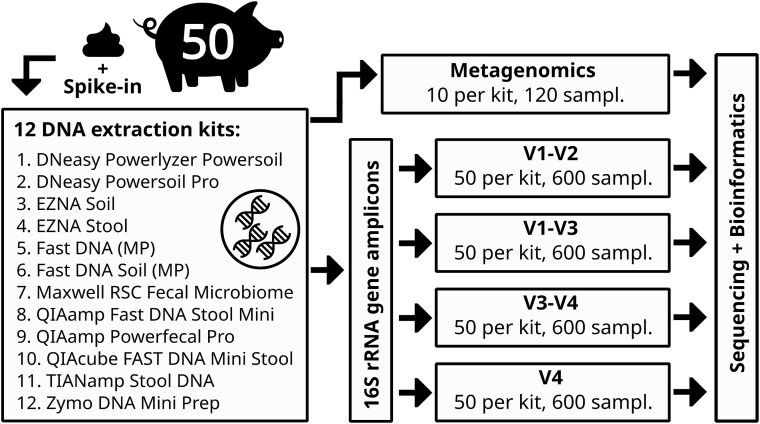
Graphical representation of the experimental setup. DNA from 50 pig fecal samples with added spike-in (ZymoBIOMICS Spike-in Control I) was extracted with 12 extraction kits. DNA was further sequenced with shotgun metagenomics (10 samples per kit) and 16S rRNA amplicon sequencing, targeting V1–V2, V1–V3, V3–V4, and V4 regions.

Fecal samples were collected from 50 male German Landrace x Pietrain crossbred pigs (Fig. 1) housed at the Agricultural Experimental Station of the University of Hohenheim (Unterer Lindenhof, Eningen, Germany), under the animal permit number 541/23. Pigs were weaned at a mean age of 33 days and sampled at a mean age of 82 days. For sample collection, the pigs were guided individually into a pen, weighed, and permitted to defecate. Samples were collected immediately upon defecation, before ground contact. Samples were placed on ice and transported to the laboratory. Upon arrival, each sample was homogenized, aliquoted, and stored at −80°C.

### DNA extraction and sequencing

DNA was extracted using 12 commercial extraction kits (Table 1) according to manufacturer’s instructions (Fig. 1). Prior to DNA extraction, 25 μL of ZymoBIOMICS Spike-in Control I (High Microbial Load) [26] (Zymo Research Europe GmbH, Freiburg, Germany) was spiked into each sample. Eluted DNA concentration was measured using a NanoDrop Spectrophotometer [27] (ThermoFisher Scientific, Germany). The V1–V2 [28], V1–V3 [28, 29], V3–V4 [30], and V4 [31] regions of the 16S rRNA gene were amplified with barcode-indexed universal bacterial primers. PCR was carried out using the Takara PrimeSTAR HS DNA Polymerase kit [32] (Takara Bio Europe SAS, Saint-Germain-en-Laye, France) in 20 μl reactions containing 1 μl of template DNA, 4 μl of 5× PrimeSTAR buffer, 1.6 μl of dNTPs, 1 μl of BioStab PCR Optimizer (II) [33] (Sigma-Aldrich Chemie GmbH, Taufkirchen, Germany), 0.5 μl of each primer, 0.2 μl of PrimerSTAR HS DNA polymerase, and 11.2 μl of nuclease-free water. PCRs for each region were performed using region-specific primers under the cycling conditions listed in Table S1 (first PCR – 10 cycles, second – 15, and third – 20). A negative control in the form of nuclease-free water was included for each 96-well plate. PCR products were verified by electrophoresis on 1.5% agarose gels.

**Table 1:** DNA extraction kits.

**No**	**Full name**	**Name in the study**	**Mechanical bead-beating**
1	DNeasy Powerlyzer Powersoil	DNeasy Powerlyzer	yes
2	DNeasy Powersoil Pro	DNeasy Powersoil	yes
3	EZNA Soil	EZNA Soil	yes
4	EZNA Stool	EZNA Stool	yes
5	FastDNA Kit (MP)	FastDNA MP	yes
6	FastDNA Kit for Soil (MP)	FastDNA MP Soil	yes
7	Maxwell RSC Fecal Microbiome	Maxwell RSC FM	yes
8	QIAamp Fast DNA Stool Mini	QIAamp FDM Stool	no
9	QIAamp Powerfecal Pro	QIAamp PFP	yes
10	QIAcube with FAST DNA Mini Stool	QIAcube FDM Stool	no
11	TIANamp Stool DNA	TIANamp Stool	yes
12	Zymo DNA Mini Prep	Zymo DNA Mini	yes

PCR libraries were normalized using the SequalPrep Normalization Plate Kit, 96-well [34] (ThermoFisher Scientific, Germany), and purified using the MinElute PCR Purification Kit [35] (Qiagen, Hilden, Germany). The DNA concentration was determined using a Qubit Invitrogen Fluorometer (ThermoFisher Scientific, Germany) with the Quantifluor dsDNA System [36] (Promega, Walldorf, Germany). The libraries were pooled in equimolar amounts and sequenced (250 × 2 bp) on an Illumina Novaseq (Illumina, San Diego, CA, USA).

DNA from 10 samples across all 12 DNA extraction protocols was subjected to shotgun metagenomic (MG) sequencing. The MG libraries were prepared from 30 μl of the final extracted DNA volume per sample, with genomic DNA concentrations ≥20 ng/μl, and sequenced using paired-end 150 bp reads with PCR-free WGS/PCR-free shotgun-based metagenomics on an Illumina Novaseq (Illumina, San Diego, CA, USA).

### Bioinformatics and statistical analyses

The 16S rRNA gene amplicon sequences were imported into Qiime2 [37], separately for each region and sequencing run. Primers were removed using cutadapt [38]. DADA2 [39] was used for denoising, merging, and chimera detection. To ensure compatibility between sequencing runs, DADA2 was employed with identical parameters for each sequencing run per targeted region. Taxonomy annotation was performed using VSEARCH-based consensus [40] and pre-fitted sklearn-based [41] full-length classifiers trained on the Silva database [42] (v138.2, 99%). Database files were processed with RESCRIPt [43].

MG samples were quality-controlled (QC) with Trim-Galore [44] (v0.6). Read quality was assessed with FastQC [45] before and after the QC step. Reads aligned to the host (GCA_000003025.6_Sscrofa11.1) and partially to the feed (GCA_902167145.1_Zm-B73) genomes were removed by Bowtie2 [46] (v2.5.3). Metagenomes were assembled for each sample using SPAdes [47] and assessed with QUAST [48]. Binning was performed by COMEbin [49]. Evaluation of bins’ “completeness” and “contamination” was carried out by BUSCO [50]. Bins and cleaned samples were imported to the Qiime2 MOSHPIT distribution [51]. Bins with ≥50% “completeness” and < 20% “contamination” were used for dereplication among all the MG samples. After dereplication, the longest bin was selected and counted as a MAG (metagenome-assembled genome). Taxonomic annotation of MAGs and cleaned samples (“read-based”) was performed using Kraken2 [52] against the “core_nt” database (v06.09.2025), with a confidence score of 0.1. Taxa abundances were estimated with Bracken2 [53]. For diversity metrics, differential abundance test, composition evaluation and core-microbiome analyses taxonomy annotation received from the “read-based” approach was used. For alpha- and beta-diversity calculations, all reads not annotated as bacterial were removed. Similarly, all MAGs not annotated as “Bacteria” at the domain level were removed before functional annotation and abundance (in “total per million”, or tpm values) estimation. Functional annotations were performed with EggNOG mapper [54] on MAGs using HMMER [55] and DIAMOND [56]. High-quality MAGs were defined as those with ≥90% “completeness” and < 5% “contamination.”

Alpha and beta diversity metrics were calculated using q2-boots [57] with bootstrapping (n = 100). Statistical analyses of alpha-diversity indices were performed using the Wilcoxon test for dependent samples [58], and beta-diversity distances were tested using the Adonis test [59]. Taxonomy profiles of 16S rRNA gene amplicon-based datasets were compared with those of MG samples for each DNA extraction kit at the genus level using the q2-evaluate-composition [60] plugin. For this purpose, accuracy metrics, including observed-to-expected taxa ratio (OET), taxonomy accuracy rate (TAR; the fraction of observed taxa that were expected) and taxonomy detection rate (TDR; the fraction of expected taxa that are observed) were calculated. Numeric values, such as ratios, counts, and percentages, that were compared between DNA extraction kits, were analyzed using Kruskal-Wallis [61] or ANOVA [62] general tests, followed by Wilcoxon or t-tests [63] for dependent samples. All *P*-values obtained from multiple comparisons were adjusted using the Benjamini-Hochberg procedure [64]. Statistical differences were denoted and plotted with the compact letter display package cld4py [65]. Differentially abundant taxa (at genus or species levels, and with relative abundance and prevalence ≥1%) were detected by Ancom-BC2 [66]. Core microbiota at the genus or species level included taxa with relative abundance ≥1% and prevalence ≥95% within the given sample group.

## Results

### DNA concentration and purity

**Figure 2:**
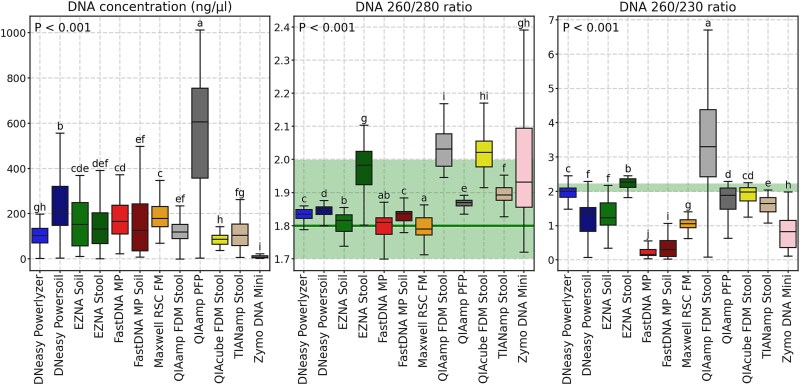
DNA concentration and purity. Boxplots representing DNA concentration in ng/ng/μl and 260/280 and 260/230 ratios across DNA extraction kits. *P*-values of Kruskal-Wallis general test are plotted in the upper part of the subplots. Letters indicate significant differences based on the adjusted *P*-values from the Wilcoxon test for dependent samples (*P*-adj < 0.05 for groups with different letters and *P*-adj ≥ 0.05 for groups with shared letters) and sorted by the median (DNA concentration and 260/230 ratio in descending order and 260/280 ratio in ascending).

We evaluated the effect of the kit on DNA concentration and purity (Fig. 2), using the 260/280 and 260/230 ratios, and both metrics differed significantly between kits.

The highest DNA concentration (ng/μl) was achieved with QIAamp PFP [median (IQR) = 606.4 (398.3); all *P*-adj < 0.05], followed by DNeasy Powersoil [212.2 (172.6); all *P*-adj < 0.05] and Maxwell RSC FM (179.2 (89.5)). The lowest DNA concentration was obtained with Zymo DNA Mini [7.5 (8.4); all *P*-adj < 0.05].

Among all the kits, Maxwell RSC FM (1.79 (0.05)), FastDNA MP (1.81 (0.05)) and EZNA Soil (1.816 (0.05)) demonstrated median 260/280 values closest to 1.8, the ratio generally considered to indicate pure DNA (Fig. 2). Other kits, such as FastDNA MP Soil (1.836 (0.03)), DNeasy Powerlyzer (1.835 (0.03)), DNeasy Powersoil (1.849 (0.02)), QIAamp PFP (1.87 (0.02)), and TIANamp Stool (1.893 (0.04)), also yielded median values close to 1.8, with most of the samples within the accepted range from 1.7 to 2. Four kits, EZNA Stool (1.982 (0.24)), QIAamp FDM Stool (2.031 (0.1)), QIAcube FDM Stool (2.021 (0.08)), and Zymo DNA Mini (1.932 (0.24)) had a median value or a relatively large fraction of samples with a ratio higher than 2.

Regarding 260/230 ratios, DNeasy Powerlyzer (1.997 (0.27)), QIAcube FDM Stool (1.978 (0.34)) and EZNA Stool (2.265 (0.25)), demonstrated median values close to the optimal range (2–2.2), followed by QIAamp PFP (1.883 (0.62)) (Fig. 2). Other kits exhibited stronger deviations.

### Effect of the 16S rRNA gene region on DADA2 performance

DADA2 output was affected by the 16S rRNA gene region. Since the V1-V3 region reads, as expected, failed to merge due to their length and the lack of an overlapping region, the DADA2 run was repeated for these reads, joining the reads instead of merging (Fig. S1A). The highest percentage of merged reads was observed for the V4 region [median (IQR) = 94.96 (2.31)], followed by the joined V1–V3 region (93.84 (2.28)). The lowest merging occurred in V1–V3 reads (3.02 (3.82)). The lowest percentages of chimeric reads were detected for V4 (1.4 (1.57)) and merged V1–V3 (1.67 (4.76)) regions, followed by V1–V2 (3.95 (2.34)). Joined V1–V3 reads exhibited the highest percentage of chimeras (16.88 (9.9)). Regarding the percentage of reads retained after DADA2, it was the highest for the V4 region (93.61 (3.27)), followed by V1–V2 (85.26 (3.17)).

The V4 region yielded the lowest number of unique ASVs (2755) (Fig. S1B). This number was higher for the merged V3–V4 (5267) and V1–V2 (8068) groups. The joined V1–V3 region produced an exceptionally high number of unique ASVs (20860). Based on these results, all samples from the V1–V3 region were excluded due to either poor merging efficiency or inflated ASVs variability introduced by paired reads joining.

### Effect of the DNA extraction kit on the quality of metagenomic samples

We also evaluated the effect of the kit on the percentage of reads retained in the MG samples after QC and the sequencing depth of cleaned samples (before the host/feed DNA removal). Most kits performed similarly (Fig. S1C), with the majority of samples losing no ˃0.5%. The exceptions were the QIAcube FDM Stool and Zymo DNA Mini kits, in which the percentage of retained reads for some samples dropped below 98%. The same kits yielded median sequencing depths notably below 3 × 10^7^.

### Taxonomy annotation of reads and DA tests

Across amplicon samples, the highest proportion of archaeal reads was found in V4 samples (up to 16%) and V3–V4 samples (up to 6%), whereas V1–V2 yielded almost exclusively bacterial assignments (Fig. S2A). Amplicon samples also contained sequences (<0.05 for V1–V2, <0.3% for V4 and < 0.6% for V3–V4) that were not annotated within any domains.

In MG samples, no effect of the kit on the proportions of *Archaea* and reads grouped into the “Others” category was observed. However, a relatively high proportion of reads were unassigned (up to 50%) (Fig. S2B). This proportion varied significantly across kits, with the lowest values observed for the Zymo DNA Mini [median (IQR) = 29.52 (8.6)]. The same kit also assigned the lowest percentage of reads to *Eukaryota* (10.11 (2.81)) and *Viruses* (2.1 (0.63)), and the highest to *Bacteria* (53.2 (13.49)). Though significant variations for the same domains were also observed between other kits, they were less pronounced as for Zymo DNA Mini. The Zymo DNA Mini also yielded the highest *Bacillota*-to-*Bacteroidota* ratios, regardless of sequencing approach or 16S rRNA gene region (Fig. S2C).

**Figure 3:**
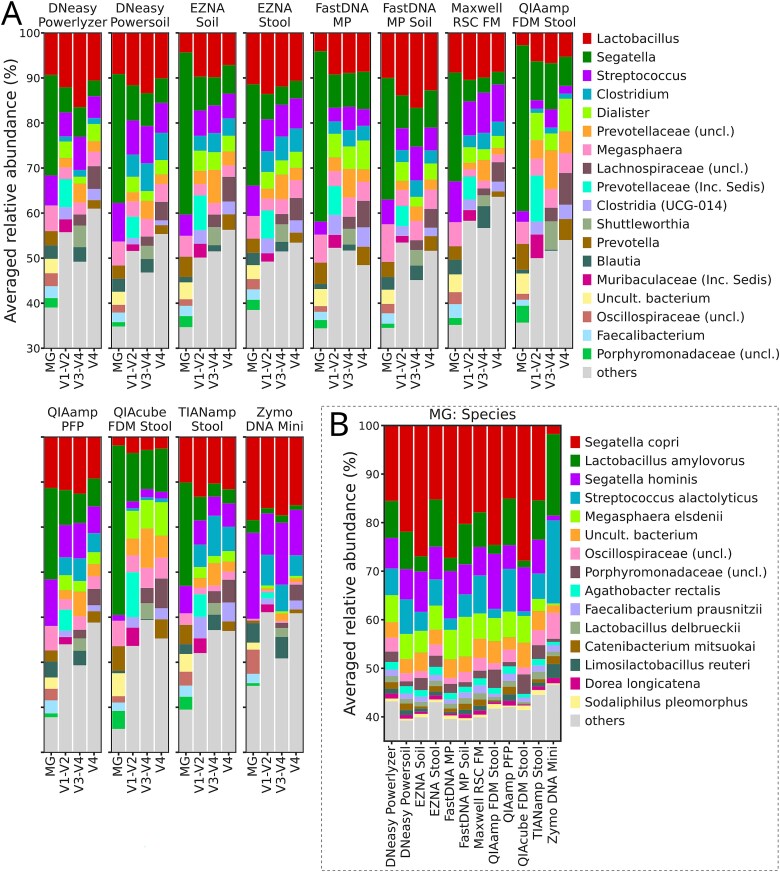
Stacked taxonomy barplots. (A) Barplots at the genus level grouped by DNA extraction kit and sequencing approach (MG for metagenomics). This subplot is split into two rows. (B) Barplots at species level (only metagenomic samples), grouped by DNA extraction kit.

We also visualized the taxonomy profiles of MG and amplicon samples within each DNA extraction kit (top 10). At the genus level, *Segatella* abundances were the highest in MG samples, except in Zymo DNA Mini kit samples, where *Streptococcus* and *Lactobacillus* dominated (Fig. 3A). In amplicon samples, the *Lactobacillus* genus was the most abundant, excluding QIAamp FDM Stool and QIAcube FDM Stool, where the dominance shifted toward *Segatella*. Relatively abundant in amplicon samples, unclassified *Prevotellaceae* was most likely annotated in the MG samples as *Prevotella*.

Differences between DNA extraction kits were also confirmed at the species level (Fig. 3B). Gram-negative bacteria *Segatella copri, Segatella hominis,* and *Megasphaera elsdenii*, abundant in MG samples, were underrepresented in the Zymo DNA mini kit, where gram-positive *Streptococcus alactolyticus* and *Lactobacillus amylovorus* dominated.

**Figure 4:**
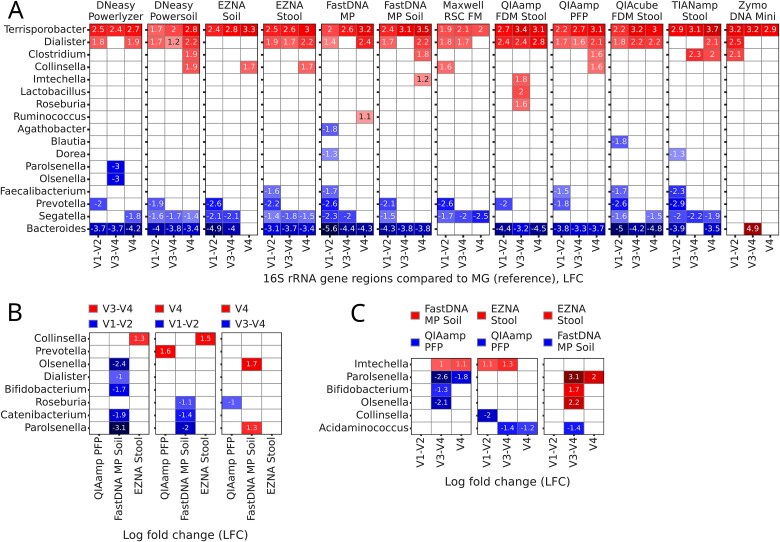
Differentially abundant taxa. Differentially abundant taxa at the genus or lower level were detected with Ancom-BC2. Log fold change (LFC) values of differentially abundant taxa (*P*-adj < 0.05, absolute LFC ≥ 1) are plotted as a heatmap. Positive values and red color indicate taxa, more abundant in the compared group and negative values and blue color - more abundant in the reference. (A) Amplicon samples (x-axis labels) compared to MG (reference) samples by DNA extraction kit (subplots). (B) 16S rRNA gene regions (legend) of amplicon samples compared to each other within the subset of DNA extraction kits (x-axis labels). (C) Comparisons of the DNA extraction kits (legend) within the subset by 16S rRNA gene region (x-axis labels).

A differential abundance test (Ancom-BC2) was performed at the genus level on features with a relative abundance of ≥1%, comparing taxonomy profiles of MG samples (as reference) with those of amplicon samples (Fig. 4A). Taxa count tables were filtered to remove unmatched classifications, retaining only taxa shared across all regions and MG samples. *Segatella* and *Bacteroides* were systematically more abundant in MG samples for most kit and region combinations. The abundances of *Terrisporobacter* and *Dialister* genera were consistently higher in amplicon samples. The Zymo DNA Mini kit identified the fewest differentially abundant taxa between MG and amplicon samples, reporting no taxa that were more abundant in MG.

Given that multiple kits from the companies Qiagen, MP Biomedicals, and Omega Bio-tek were utilized in this study, we identified the best-performing kit from each company based on DNA concentration and purity, optimal ratio of microbial spike-in recovery, MAGs quality, and percentage of bacterial reads annotated from MG data. Accordingly, we selected three kits, QIAamp PFP (Qiagen), FastDNA MP Soil (MP Biomedicals), and EZNA Stool (Omega Bio-tek), for pairwise comparisons of 16S rRNA gene regions (Fig. 4B) and kits (Fig. 4C). In samples extracted with the FastDNA MP Soil kit, abundances of *Parolsenella* and *Catenibacterium* were higher when targeting V1–V2 region compared to both V3–V4 and V4 (Fig. 4B). In the same kit/region combination, abundances of *Olsenella*, *Dialister,* and *Bifidobacterium* were higher compared to V3–V4 and *Roseburia* when compared to V4. FastDNA MP also resulted in higher *Olsenella* and *Parolsenella* abundances in V4 samples than in V3–V4. Two genera were reported as differentially abundant between regions when QIAamp PFP was used for DNA extractions – *Prevotella* was more abundant in V4 samples compared to V1–V2 and *Roseburia* was more abundant in V3–V4 samples compared to V4. Regarding the EZNA Stool kit, among all comparisons, only *Collinsella* was differentially abundant, with higher abundances in the V3–V4 and V4 regions than in the V1–V2.

When comparing the subset of kits in the pairwise mode by each region (Fig. 4C), V3–V4 samples yielded the highest number of differentially abundant taxa. *Parolsenella, Bifidobacterium,* and *Olsenella* abundances were lower when extracted with FastDNA MP Soil kit compared to QIAamp PFP and EZNA Stool. V1–V2 samples demonstrated no differences between the FastDNA MP kit and the same kits as above. QIAamp PFP kit resulted in lower abundances of *Imtechella* (spike-in) compared to FastDNA MP Soil (V3–V4 and V4 regions) and EZNA Stool (V1–V2 and V3–V4 regions) kits. Finally, EZNA Stool kit extractions yielded lower abundances of *Collinsella* compared to QIAamp PFP in V1–V2 samples and higher abundances of *Parolsenella* compared to FastDNA MP Soil samples in V4 samples.

### Composition, diversity, and taxonomy annotations of MG and 16S rRNA gene samples

**Figure 5:**
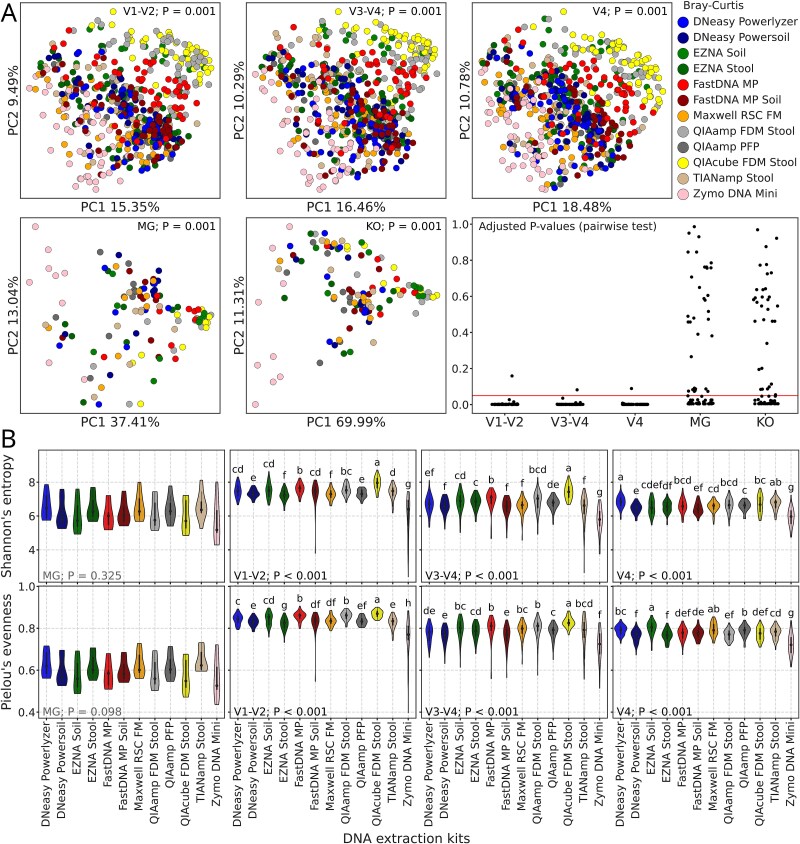
Composition and diversity of MG and amplicon samples. (A) PCoA plots based on Bray-Curtis distances for amplicon (upper panel) and MG samples (bottom panel, left), KO functional annotations of MG samples (bottom panel, middle) and dotplots with *P*-adjusted values from Adonis pairwise comparisons between DNA extraction kits for each group of samples (bottom panel, right), with the horizontal red line indicating significance threshold (0.05). *P*-values of Adonis general test are plotted in the upper part of the subplots. (B) Violin plots representing alpha diversity metrics (Shannon’s entropy and Pielou’s evenness) of MG and amplicon samples. *P*-values of Kruskal-Wallis general test are plotted at the bottom of the subplots. Letters indicate significant differences based on the adjusted *P*-values from the Wilcoxon test for dependent samples (*P*-adj < 0.05 for groups with different letters and *P*-adj ≥ 0.05 for groups with shared letters) and sorted by the median in descending order.

The bacterial community of MG and amplicon samples, as well as the functional profiles (MG), were affected (all *P*-values <.01) by the kit (Fig. 5A). Almost all pairwise comparisons between the kits were significant for each 16S rRNA gene regions, while approximately half of the comparisons were insignificant for MG samples and their functions.

Alpha diversity metrics (Shannon’s entropy and Pielou’s evenness), were affected by the kit in amplicon samples (all *P*-values from the general test <.001), but not in MG samples (all *P*-values from the general test >.05) (Fig. 5B).

**Figure 6:**
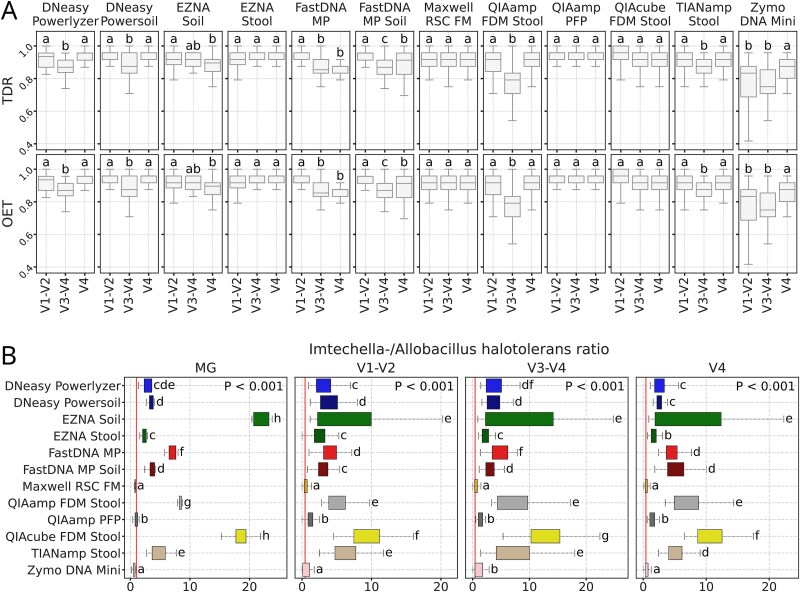
Evaluation of taxonomy profiles similarities between MG and amplicon samples and Spike-In ratios. (A) Taxonomy detection rate (TDR) and observed-to-expected taxa ratio (OET) of amplicon samples compared to averaged MG samples by each DNA extraction kit. Letters indicate significant differences based on the adjusted *P*-values from the paired t-tests (*P*-adj < 0.05 for groups with different letters and *P*-adj ≥ 0.05 for groups with shared letters) and sorted by the mean in descending order. (B) *Imtechella halotolerans/Allobacillus halotolerans* ratios for MG (baseline 1) and amplicon (baseline 0.43) samples. *P*-values of Kruskal-Wallis general test are plotted at the bottom of the subplots. Letters indicate significant differences based on the adjusted *P*-values from the Wilcoxon test for dependent samples (*P*-adj < 0.05 for groups with different letters and *P*-adj ≥ 0.05 for groups with shared letters) and sorted by the median in ascending order.

To investigate which of the 16S rRNA gene regions produced taxonomy profiles most similar to those of MG samples, taxonomy accuracy and detection rates (TAR and TDR), as well as observed-to-expected taxa (OET), were calculated at the genus level (Fig. 6A). Regardless of the kit and 16S rRNA region, all TAR values were equal to the optimal value of 1. TDR and OET values for the V1–V2 and V4 regions were higher across most kits (P-adj < 0.05) than for V3–V4.

### Spike-in detection and ratios

ZymoBIOMICS Spike-in Control I was used as a spike-in during DNA extraction of all samples. We estimated that the theoretical ratio (hereafter “I/A ratio”) of two species included in the spike-in standard, *Imtechella halotolerans* (Gram-negative) and *Allobacillus halotolerans* (Gram-positive), should be 1 (1:1) for MG (by the number of genome copies) and 0.43 (3:7) for amplicon samples (by the number of 16S rRNA gene copies).

Among MG samples, the closest I/A ratios were achieved with QIAamp PFP [median (IQR) = 1.034 (0.424)], closely followed by Maxwell RSC FM (0.781 (0.074)) and Zymo DNA Mini (0.716 (0.239)) (Fig. 6B). Regarding amplicon samples, the closest to theoretical I/A ratios were observed in Maxwell RSC FM [V4: 0.463 (0.315), V1–V2: 0.481 (0.428), V3–V4: 0.549 (0.465)], followed by Zymo DNA Mini [V1–V2: 0.605 (0.984), V4: 0.653 (0.414), V3-V4: 1.132 (1.339)] and QIAamp PFP [V1–V2: 1.172 (0.682), V3–V4: 1.258 (0.752), V4: 1.26 (0.763)].

### Bacterial core microbiome

The bacterial core microbiome at the genus or species (MG) level was defined as taxa detected in at least 95% of samples within a specific group (defined by sequencing approach or region and DNA extraction kit), with an additional threshold of an average relative abundance ≥1%.

Among the MG samples, most kits yielded 30 core species (Fig. S3A). The EZNA Soil, QIAamp FDM Stool, and QIAcube FDM Stool kits lacked *Blautia massiliensis*. The same species and *Phascolarctobacterium succinatutens* were missing among the core species of the TIANamp Stool kit. At the genus level, all kits with MG samples performed equally, reporting 33 core genera, except for the TIANamp Stool kit, which failed to report *Selenomonas*, *Phascolarctobacterium*, *Bacteroides,* and *Streptomyces* (Fig. S3A and B).

The core genera of amplicon samples varied among regions and kits (Fig. S3A and B). In V1–V2 samples, the highest number of core genera (33) was obtained with FastDNA MP and QIAamp PFP, and the lowest (12) with Zymo DNA Mini. With V3–V4, the top two “performers” among the kits were QIAcube FDM Stool (33) and QIAamp PFP (33), while for QIAamp FDM Stool and TIANamp Stool the number of core genera was ˂20. The DNeasy Powerlyzer and QIAamp PFP kit yielded the best results with V4 samples, identifying 32 core genera. The lowest numbers in V4 samples were observed for Zymo DNA Mini (23) and EZNA Soil (23).

### Effect of the DNA extraction kit on MG assemblies and MAGs binning

**Figure 7:**
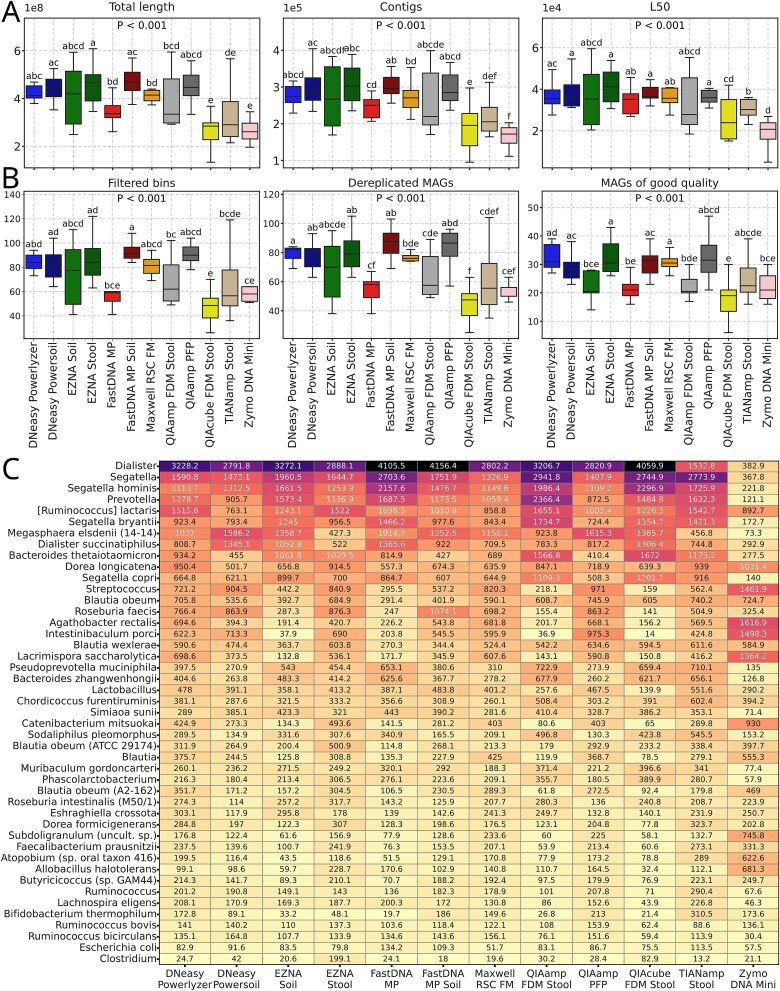
MG assemblies quality and MAGs reconstruction. (A) Assemblies quality according to the QUAST reports: Total length of assemblies (×108), number of contigs (×105), and L50 values (×104). (B) Number of binned MAGs that were retained after filtering (“completeness” ≥ 50% and “contamination” < 20%), number of MAGs after dereplication and number of MAGs of good quality (“completeness” ≥ 90% and “contamination” < 5%). (C) TPM values of MAGs of good quality (“completeness” ≥ 90% and “contamination” < 5%) which were annotated to genus or species level. In all plots, *P*-values of Kruskal-Wallis general test are plotted in the upper part of the subplots. Letters indicate significant differences based on the adjusted *P*-values from the Wilcoxon test for dependent samples (*P*-adj < 0.05 for groups with different letters and *P*-adj ≥ 0.05 for groups with shared letters) and sorted by the median in descending order.

Individual assemblies were constructed for each MG sample with SPAdes. The total length of assemblies, the number of contigs, and the L50 values showed consistent trends across DNA extraction kits (Fig. 7A). The most striking differences were observed for Zymo DNA Mini, QIAcube FDM Stool, and, to a lesser extent, TIANamp Stool, with the lowest metric values.

After binning, all bins were filtered based on completeness (≥ 50%) and contamination (< 20%) thresholds (Fig. 7B). The retained bins were used for dereplication and referred to as MAGs. Good quality MAGs were defined as MAGs with completeness ≥90% and contamination <5%. The highest numbers of dereplicated and good-quality MAGs were obtained using the DNeasy Powerlyzer and Powersoil, EZNA Stool, FastDNA MP Soil, Maxwell RSC FM, and QIAamp PFP kits. In contrast, the lowest numbers were observed with the QIAcube FDM Stool, TIANamp Stool, Zymo DNA Mini, FastDNA MP, and QIAamp FDM Stool kits.

Averaged total per million (tpm) values of high-quality MAGs that were successfully annotated at least to the genus level were plotted as a heatmap (Fig. 7C). The most distinct profile of high-quality MAG abundances was shown by the Zymo DNA Mini kit, in which the *Dialister*, *Segatella,* and *Prevotella* genera and species, which were relatively abundant in samples extracted with other kits, were less represented. In contrast, *Streptococcus, Agathobacter rectalis, Intestinobaculum porci,* and *Lacrimispora saccharolytica* had higher proportions.

## Discussion

This study provides a comprehensive benchmark of 12 commercially available DNA extraction kits across both MG and 16S rRNA gene amplicon sequencing approaches in the pig gut microbiome, a model system of agricultural and biomedical relevance [67–69]. By jointly evaluating sequencing strategies and targeted 16S rRNA gene hypervariable regions, we demonstrated that DNA extraction protocols introduce systematic biases, with implications for reproducibility in microbiome science. To achieve this, fecal samples from 50 piglets were used for DNA extraction, yielding 120 MG samples (10 per kit) and ~2400 amplicon samples (50 per kit). We assessed how kit and region choice influenced DNA yield and purity, taxonomical and functional profiles, diversity metrics and core microbiome, as well as its impact on metagenomic assemblies and MAGs. Moreover, we used ZymoBIOMICS Spike-in to calculate the extent to which observed gram-negative and gram-positive bacteria ratios matched theoretical expectations.

Among all kits, the top three by DNA yield were QIAamp PFP, DNeasy Powersoil, and Maxwell RSC FM, with a drastic difference between QIAamp PFP and the closest top performer. A relatively high DNA concentration, using the same kit, has also been reported for human fecal samples [70]. The performance of two kits (QIAamp FDM Stool and QIAcube FDM Stool), which lacked a mechanical bead-beating step, was lower than that of top bead-beating kits and consistently outperformed only the Zymo DNA Mini, which produced the lowest concentrations. Regarding DNA purity, four kits, two without bead-beating (QIAamp FDM Stool and QIAcube FDM Stool) and two with bead-beating (EZNA Stool and Zymo DNA Mini), yielded 260/280 ratios above the recommended 1.7–2 range for a subset of samples, indicating the possible presence of RNA residuals [71, 72].

We investigated the effect of 16S rRNA gene region on DADA2 output. Since primers may introduce artificial variability into ASVs through degeneracy [73, 74], we used sequences that were already trimmed of primers. As expected from the lengths of the targeted regions and the sequencing length (250 × 2), reads from the V1–V3 subset failed to merge. In similar cases, researchers typically either discard reverse or (rarely) forward reads or join paired reads. Although direct joining has been shown to improve taxonomic classification compared with using only forward or reverse reads [75], our data indicate that this approach yields a high proportion of chimeric sequences and unique ASVs, potentially affecting diversity metrics. Given that the V1 region alone, unlike the V4 region, is not commonly used, and that forward reads of the V1–V3 region were sequenced with the same primer as the V1–V2 region, we excluded the V1–V3 region from downstream analysis rather than rely on forward-only reads. We recommend using the V1–V3 region only when sequenced using either Illumina 300 × 2 (not tested within the study) or long-read platforms. Regarding the remaining 16S rRNA gene regions, the highest percentage of merged reads was observed for V4 amplicons, followed by the V3–V4 region. The number of unique ASVs (frequency ≥ 10, present in ≥2 samples) was higher in the DADA2 output from the V1–V2 region than in those from the V4 and V3–V4 regions, suggesting greater variability in the V1–V2 region within bacterial communities.

For MG samples, we evaluated the kit’s effect on raw read quality control by calculating the percentage of retained reads. Despite some variability, the resulting percentages for most samples were above 99.5%. Two kits, the Zymo DNA Mini and QIAcube FDM Stool, demonstrated the highest read loss. However, these values did not exceed 2% and are unlikely to affect downstream analyses. More pronounced differences between kits were observed in sequencing depth, with QIAamp PFP, EZNA Soil and FastDNA MP producing approximately double the median depth compared to the Zymo DNA Mini kit. Nevertheless, Zymo DNA Mini yielded the highest proportion of bacterial reads among total reads in downstream analyses, despite its lower sequencing depth.

After taxonomy annotation, differences among amplicon samples were apparent. Almost all V1–V2 reads were classified as *Bacteria*, consistent with reports that this region reduces off-target amplification [76]. The V3–V4 and V4 subsets contained up to 16% archaeal reads and a small portion of unassigned reads, presumably due to non-specific amplification [76, 77]. At the same time, no effect of the DNA extraction kit on archaeal DNA yield was observed across the MG samples. By contrast, the proportions of *Eukaryota*, *Viruses*, *Bacteria,* and unassigned reads varied significantly between the kits, with the Zymo DNA Mini kit yielding the highest proportion of *Bacteria*, followed by the QIAamp PFP. Although Zymo DNA Mini kit enriched bacterial sequences, its community profiles differed markedly, with inflated *Bacillota*-to-*Bacteroidota* ratios across both MG and amplicon samples targeting all 16S rRNA gene regions.

Differences extended to species-level taxonomic resolution (read-based) and MAGs abundances in MG samples. *Segatella copri* and *Segatella hominis* were underrepresented among the read-based annotations and *Prevotella*, *Segatella* and *Staphylococcus hominis* among the MAGs had lower abundances when samples were extracted by the Zymo DNA Mini kit. Interestingly, despite these compositional changes, Zymo DNA Mini kit showed greater similarity between MG (read-based) and amplicon profiles at the genus level.

When comparing bacterial genera profiles between MG and amplicon samples, it was noted that taxonomic resolution often differed by method, database and classification tool. For example, unclassified *Prevotellaceae* in amplicon data likely corresponded to *Prevotella* in MG. Moreover, differences in achieved taxonomy resolution may exist not only between samples sequenced using different approaches, but also between amplicon samples targeting different 16S rRNA gene regions [78], due to variability in resolution. These discrepancies may introduce artificial biases into differential abundance tests by inflating the number of false zeros across all samples in one group, thereby affecting normalization or bias-correction steps [79]. Therefore, when comparing taxonomy profiles between MG and amplicon samples at the genus level by Ancom-BC2, we excluded all mismatched taxons. Our results demonstrated that, for most combinations of DNA extraction kits and targeted 16S rRNA gene regions, *Terrisporobacter* (Gram-positive) and *Dialister* (Gram-negative) were more abundant in amplicon samples, while Gram-negative *Segatella* and *Bacteroides* prevailed in metagenomic samples. Caution should be taken when interpreting the results from the differential abundance test when comparing MG and amplicon samples. We annotated MG samples using Kraken2 and Bracken, which report bacterial abundances after correction for the number of 16S rRNA gene copies [52, 53]. In contrast, no such correction was implemented for 16S rRNA gene amplicon sequencing data. However, since we were primarily interested in comparing how taxonomy profiles are reported across different sequencing approaches using the same group of animals and commonly employed practices, our results remain scientifically sound.

In general for amplicon samples, our results demonstrate that the choice of kit exerts a stronger or at least equal influence on amplicon-based microbiome profiles than hypervariable region selection alone. This is in line with prior reports emphasizing the roles of lysis protocols and extraction chemistry in shaping microbial community recovery [80–86]. Our spike-in analyses also support the findings regarding the role of the DNA extraction kit in reported bacterial communities, as the obtained I/A ratios are largely dependent on the extraction kit, yielding similar results across all targeted regions and between amplicon and MG samples. However, it should be noted that the selection of the 16S rRNA gene region or primers for amplification is often dependent on the targeted sampling niche, as they have been shown to perform differently when targeting bacterial communities across niches or hosts [87–89].

For MG, differences between kits were less pronounced: composition and functional profiles were affected, but approximately half of the pairwise comparisons were insignificant. It was previously reported that MG sequencing reduces technical variation among replicates [90]. Our findings are somewhat in line with this observation, though we demonstrate the reduced variation among replicates obtained by different DNA extraction kits. Alpha diversity metrics also differed by kit in amplicon data, but not in MG, consistent with previous findings that extraction biases disproportionately impact amplicon-based studies [91].

Core microbiome analysis further supported these findings: MG samples were largely consistent across kits, except TIANamp Stool, whereas amplicon-based cores varied substantially by kit and region, with the stronger effect of the kit. QIAamp PFP consistently recovered the largest number of core genera across all 16S regions and MG samples, thereby reinforcing its reliability.

Since our study was conducted in a single laboratory, additional investigations in the “ring-trials” format are required to assess biases introduced by different laboratories and/or handling personnel.

## Supplementary Material

02_Supplementary_Tables_Yergaliyev_Enokela_etal_ycag097

03_Supplementary_Figures_Yergaliyev_Enokela_etal_ycag097

## Data and code sharing

The datasets generated and/or analyzed during the current study are available in the European Nucleotide Archive repository (ENA) under the accession number PRJEB101201. The code used in this study is available in the project’s Git repository (https://github.com/timyerg/LimBiom_Pilot/releases/tag/v1.1.0).
