# Supplementary material for: Toward reproducible pig gut microbiome profiling through standardized methodologies
Source: ISME Commun. 2026 Apr 11;6(1):ycag097. doi: 10.1093/ismeco/ycag097 (PMC13155110; doi:10.1093/ismeco/ycag097)
Supplement: 02_Supplementary_Tables_Yergaliyev_Enokela_etal_ycag097 [file 02_supplementary_tables_yergaliyev_enokela_etal_ycag097.pdf]

Supplementary Table 1. 16S rRNA gene primers and PCR cycles.

Region	Primers	Initial denat.	Cycles			Final Extension
			Denat.	Annealing	Extension	
**V1-V2**	27F-AGRGTTHGATYMTGGCTCAG, 338R-TGCTGCCTCCCGTAGGAGT	95ºC (3 min.)	98ºC (45 sec.)	55ºC (10 sec.)	75ºC (45 sec.)	72ºC (2 min.); 4ºC (∞)
**V1-V3**	27F-AGRGTTHGATYMTGGCTCAG, 534R-ATTACCGCGGCTGCTGG			56ºC (10 sec.)		
**V3-V4**	338F-ACTCCTACGGGAGGCAGCAG, 806R-GGACTACHVGGGTWTCTAAT			55ºC (10 sec.)		
**V4**	515F-GTGCCAGCMGCCGCGGTAA, 806R-GGACTACHVGGGTWTCTAAT			50ºC (10 sec.)		
