# Supplementary material for: Toward reproducible pig gut microbiome profiling through standardized methodologies
Source: ISME Commun. 2026 Apr 11;6(1):ycag097. doi: 10.1093/ismeco/ycag097 (PMC13155110; doi:10.1093/ismeco/ycag097)
Supplement: 03_Supplementary_Figures_Yergaliyev_Enokela_etal_ycag097 [file 03_supplementary_figures_yergaliyev_enokela_etal_ycag097.pdf]

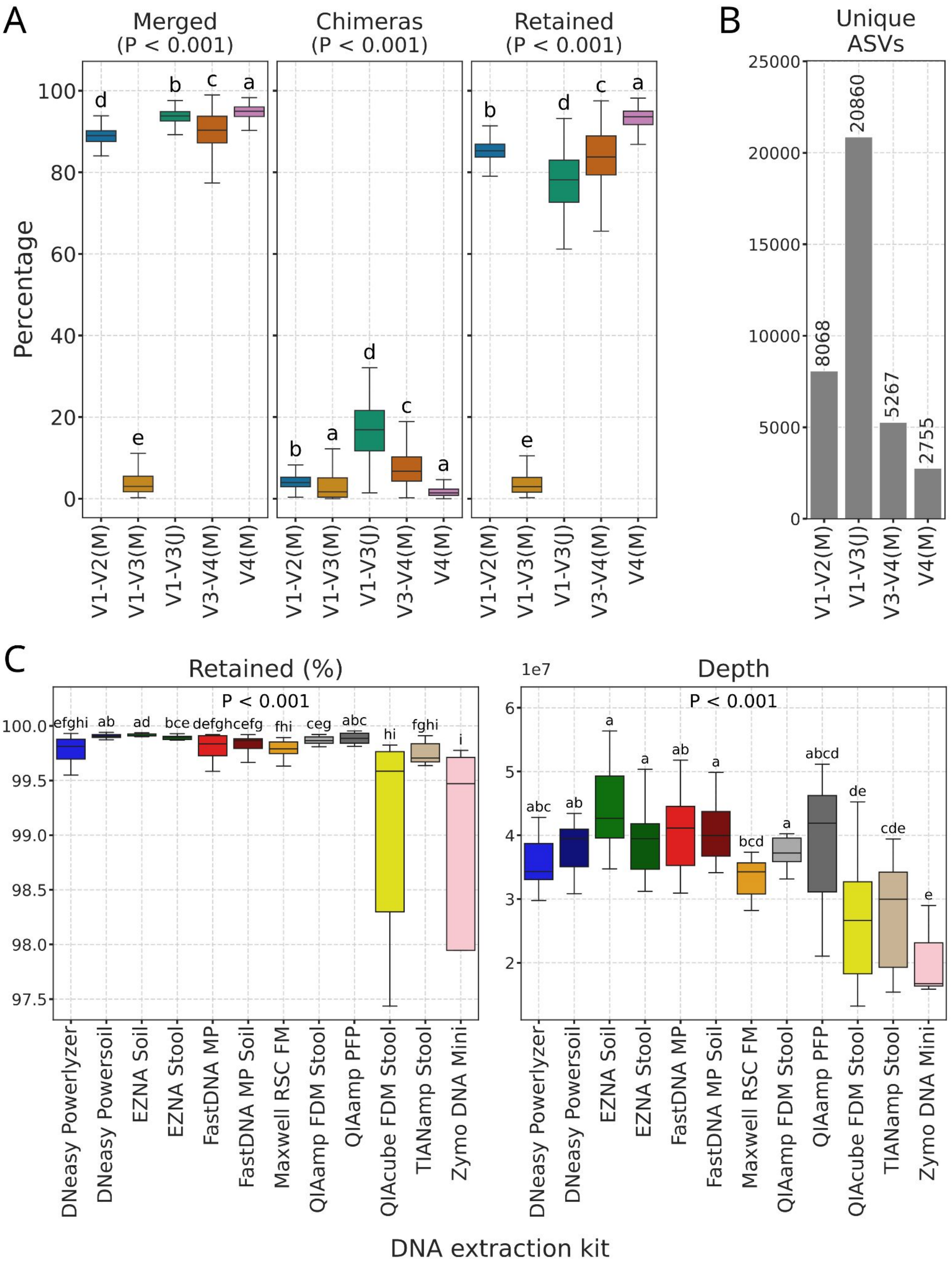


**Figure S1. Effect of the 16S rRNA gene region on DADA2 output and DNA extraction kit on QC of metagenomic samples.** A) Boxplots representing the percentage of: -i Merged - reads that were successfully merged after initial quality-based filtering; -ii Chimeras - reads that were identified as chimeric; -iii Retained - reads that passed all filtering steps and merging, for each 16S rRNA gene region. Whether reads were merged ("M") or joined ("J") is indicated on x-axis labels. B) Barplots showing the total number of unique ASVs (with total frequency ≥ 10 and that were present in at least 2 samples) in all the samples from given 16S rRNA gene region. C) Boxplots representing the following data from the MG samples: -i percentage of reads passed QC; and -ii the depth as total number of reads retained after QC (x 10 000 000) across DNA extraction kits. In all plots P-values of Kruskal-Wallis general test are plotted in the upper part of the subplots. Letters indicate significant differences based on the adjusted P-values from the Wilcoxon test for dependent samples (P-adj < 0.05 for groups with different letters and P-adj ≥ 0.05 for groups with shared letters) and sorted by the median in descending order.

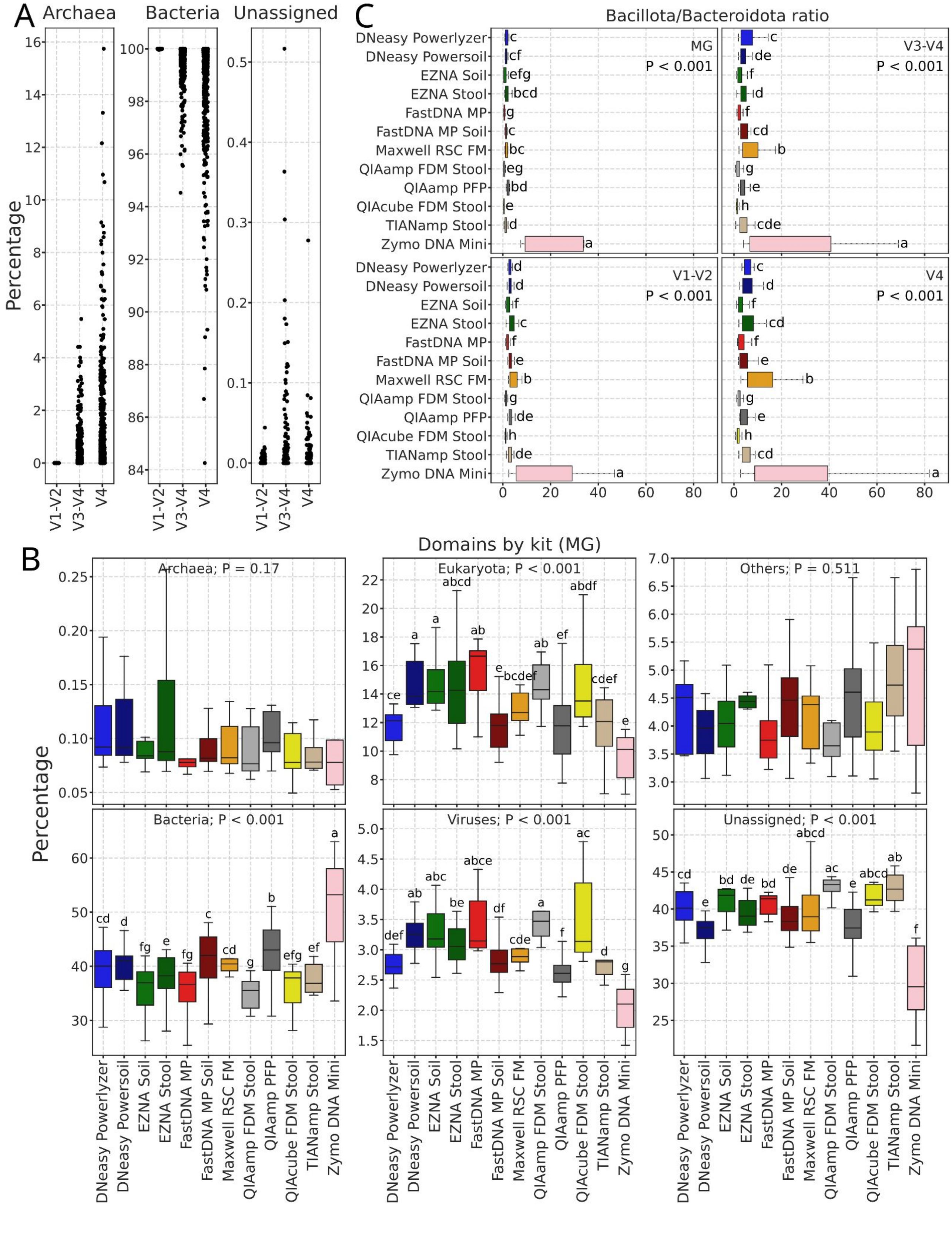

**Figure S2. Taxonomy annotation of amplicon and MG samples.** A) Dotplot representing the percentages of reads annotated at the domain to Archaea, Bacteria or unassigned. B) Boxplots representing the percentages of reads in metagenomic samples that were assigned at the domain level to Archaea, Bacteria, Eukaryota, Viruses or unassigned by DNA extraction kit. Reads that were assigned at the domain level by the "core_nt" database as "d__containing" were grouped into "Others" category. C) Boxplots representing Bacillota/Bacteroidota ratios. In all plots P-values of Kruskal-Wallis general test are plotted in the upper part of the subplots. Letters indicate significant differences based on the adjusted P-values from the Wilcoxon test for dependent samples (P-adj < 0.05 for groups with different letters and P-adj ≥ 0.05 for groups with shared letters) and sorted by the median in descending order.

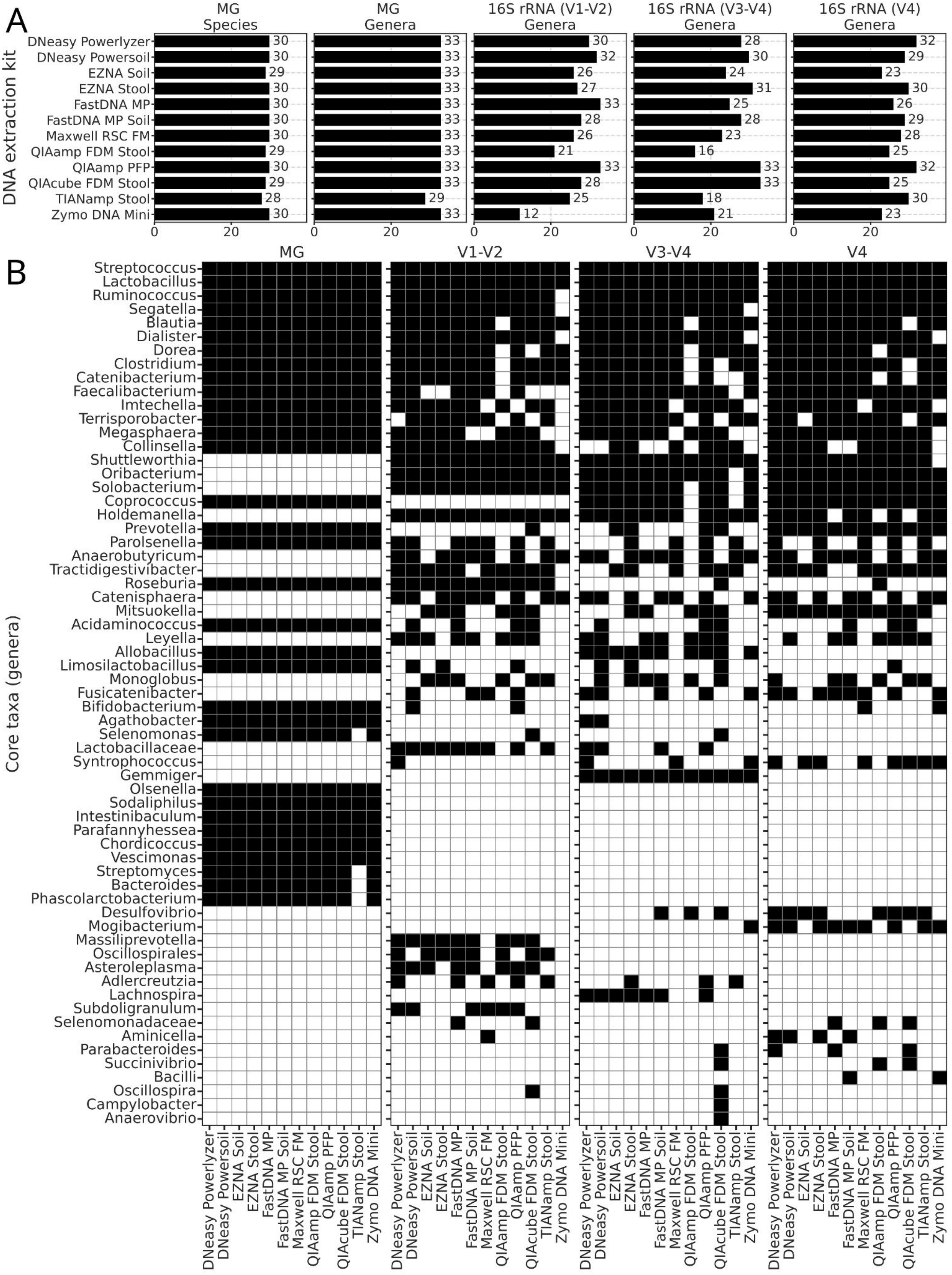


**Figure S3. Bacterial core microbiome at genus and species (MG) levels.** A) Per kit counts of core species (MG) and genera for MG an amplicon samples. B) Presence (black) or absence (white) of bacterial genera in the core microbiome by DNA extraction kit in MG and amplicon samples.
